# The challenges for targeting Chagas disease for elimination as a public health problem

**DOI:** 10.1590/0074-02760210033chgsa

**Published:** 2022-05-16

**Authors:** Guilherme Loureiro Werneck

**Affiliations:** 1 Universidade Federal do Rio de Janeiro

Chagas Disease (CD) is a neglected tropical disease restricted initially to rural areas of Latin America. Today, due to population movements and the process of urbanisation, most affected people live in Latin American urban areas. Migration has contributed to the dissemination of CD cases worldwide. Consequently, the disease has been identified in a range of non-endemic countries outside Latin America, most cases being recorded in the United States of America, Spain, Italy, and Canada. It is estimated that between 6 to 7 million people are infected with the *Trypanosoma cruzi* parasite in the world, and around 75 million people are at risk of infection, mostly in Latin American countries. In 2019, an estimated 5,500-16,500 people died from Chagas disease, and about 184,000-459,000 disability-adjusted life years were lost due to the disease worldwide.^1^

In Latin America, the number of new CD cases has been significantly reduced in the last decades, mainly due to a decrease in vector-borne and blood transfusion transmission routes, with oral transmission being responsible for most CD acute cases reported. In non-endemic countries, the main transmission routes are blood transfusion, organ transplantation, and vertical transmission.^2^

The World Health Organization (WHO) established new goals for controlling CD in both endemic and non-endemic countries by 2030, including the disease targeted for its elimination as a public health problem.^3^ The goals include the interruption of transmission through the four transmission routes (vectorial, transfusion, transplantation and congenital), with access to antiparasitic treatment covering 75% of the eligible population in 15 endemic countries; the interruption of intradomiciliary (vectorial) transmission in 86% of endemic countries; the complete interruption of blood transfusion and tissue transplant-related transmissions; and verification of interruption of congenital transmission in 15 (37%) of the targeted countries.^3^ The challenges for achieving such goals are immense but feasible. However, targeting Chagas disease for elimination involves the essential commitment that countries recognise Chagas disease as a public health problem. It will also require improving access to health care for Chagas disease and establishing intersectoral and integrated multi-country surveillance and control strategies, all rooted in evidence-based guidelines and human rights principles.^3^ ^,^ ^4^ In this context, mathematical modelling studies are essential for tracking the progress towards the new goals, particularly at finer geographical scales.^5^

In this context, the article “Emerging and reemerging forms of *Trypanosoma cruzi* transmission” by Dr Yasuda is mostly welcome, providing a comprehensive review of the significant challenges for achieving the goal of interrupting the *T. cruzi* infection transmission routes through blood transfusion, organ transplantation, maternofetal and contaminated food by 2030.^6^

Concerning the transmission by blood or blood products, the recommendations rely on the universal use of highly sensitive (≥ 99%) serological tests in endemic areas and screening targeted at high-risk groups in non-endemic regions. Although these approaches are well-founded, it should be noticed that accuracy of serological tests for detecting the *T. cruzi* infection show variability from 94% to 100%, indicating the need for a careful evaluation before acquisition and use of such tests.^7^ Additionally, reported sensitivities and specificities of diagnostic tests from producers are prone to different sources of bias, such as spectrum bias (e. g., not including samples with low antibody titers) and misclassification bias due to imperfect reference tests. Therefore, developing standardised protocols and well-designed reference panels for independent and multicentric validation of diagnostic tests is essential for adequate control of transmission by blood or blood products.

Regarding the challenges for elimination of congenital CD, Yasuda^6^ reinforces the need for an effective surveillance system for identification and diagnosing infected women of reproductive age, pregnant infected women and infected babies. As well evoked by Yasuda, this can only be achieved with robust, effective and comprehensive primary care. Therefore, it is, more than ever, fundamental the strengthening the chronic underfunded Brazilian Unified Health System (SUS). Reversing the fiscal policies and austerity measures implemented since 2016 is an immediate requirement for developing comprehensive, equitable and sustained public health policies necessary for meeting the WHO 2030 goals.

Finally, the issue of controlling oral transmission of CD is a significant challenge because it involves social, economic and environmental issues. The risk of oral transmission depends on the interfaces between the sylvatic vector transmission cycle and financial activities for community subsistence, heightened by the lack of good food processing practices.^6^ Dealing with such a transmission source needs a broad and integrated multisectoral approach that involves better surveillance, diagnostic, and care of acute Chagas disease cases and the strengthening of health educational approaches that promote the active participation of communities. Policies and regulations of practices to control food contamination are essential, but alone might contribute to worsening the economic and social situations of already vulnerable populations. Therefore, investment in safe food production practices within the solidarity economy framework and considering the specificities of the social and cultural context is an alternative to guarantee food safety, reducing the risk of oral transmission of CD, and employment and income for that population.

There were significant signs of progress in the control of CD over the last decades, but much remain to be done. On the one hand, it is essential to reinforce and expand the coverage and effectiveness of health surveillance programs to avoid the risk of post-honeymoon disease re-emergence due to the well-known tendency for downscaling and discontinuation of programs after successful control. At the same time, it is necessary to warrant adequate access to qualified health care, develop new and better diagnostic tests and clinical protocols, and invest in creative and economic sustainable actions to foster the engagement of the community in the control and prevention of CD transmission.

Comments on the article: Shikanai-Yasuda MA. Emerging and reemerging forms of *Trypanosoma cruzi* transmission. Mem Inst Oswaldo Cruz. 2022; 117: e210033.
